# Notes from the Editor

**Published:** 2010-02

## 2009 Reviewers of the Year

Like all peer-reviewed journals, *EHP* relies on the diligence and integrity of experts to help determine the quality and impact of papers submitted for possible publication. In 2009 *EHP* received nearly 1,200 papers, and about 480 of those papers were sent by our Associate Editors to two to four anonymous peer reviewers for evaluation. *EHP* published 292 papers in 12 issues during 2009, and the journal is very grateful for the time and effort of the more than 1,000 reviewers who assisted us last year. A list of those reviewers is available on the journal’s website (http://ehponline.org/article/info:doi/10.1289/ehp.118-a59).

For the first time, *EHP* is recognizing it’s top 12 Reviewers of the Year, individuals who reviewed at least six papers during the year and received excellent ratings for the timeliness and quality of their reviews by the Associate Editor who handled the peer-review process. They are John Balbus, David Carpenter, Deborah Cory-Slechta, Amy Herring, William Kelce, Morton Lippmann, Matthew Longnecker, Arnold Schecter, Rémy Slama, David Thomas, Leo Trasande, and Mary Wolff. We congratulate these reviewers and thank the hundreds of others who contributed to the success of *EHP* in 2009.

## Revised Instructions for Authors for 2010

Authors planning to submit manuscripts to *EHP* should note that we have revised our Instructions to Authors (ITA). The revised ITA can be found in this issue (p. A83) and are available online (http://www.ehponline.org/static/instructions.action). Authors should note that the word limit for Letters to the Editor has been reduced from 1,000 to 750 words, and we have tried to make it clear that it is not acceptable to introduce new non–peer-reviewed data in Letters to the Editor. In addition, *EHP* reserves the right not to publish letters deemed by the editors to be overly personal or polemic.

The revised ITA provide more detailed information concerning scientific integrity. Authors should be aware that *EHP* now routinely evaluates manuscripts for possible plagiarism. The revised ITA also emphasize our policy of full disclosure of all actual or potential financial or nonfinancial competing interests involving people or organizations that might reasonably be perceived as relevant. Authors must evaluate wording in documents such as grants or contracts to determine whether there might be an actual or potential competing interest. Another major change in the ITA is that *EHP* will actively evaluate submissions for research that might provide knowledge, products, or technologies that could be directly misapplied to pose a threat to public health and safety.

Other revisions in the ITA include minor changes in guidance for preparation of manuscripts, including tables and figures; updated information concerning the online submission process; and a new embargo policy. In addition, we now recommend that authors refer to the *AMA Manual of Style: A Guide for Authors and Editors* in preparing manuscripts. Questions concerning changes in the revised ITA should be directed to EHPmanuscripts@niehs.nih.gov.

## Figures and Tables

**Figure:**
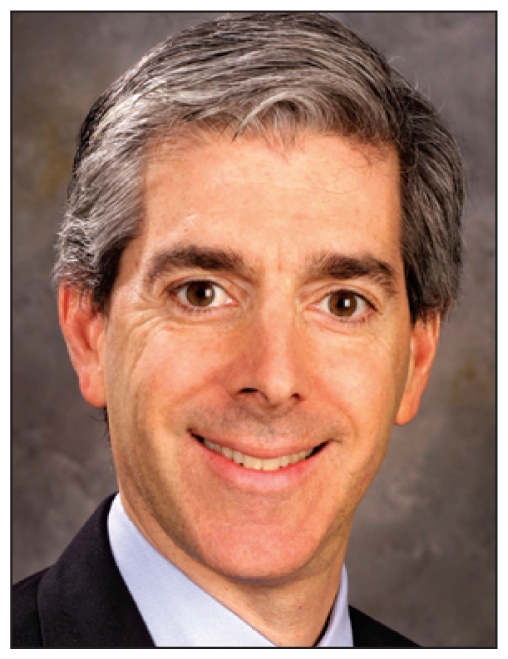
John Balbus

**Figure:**
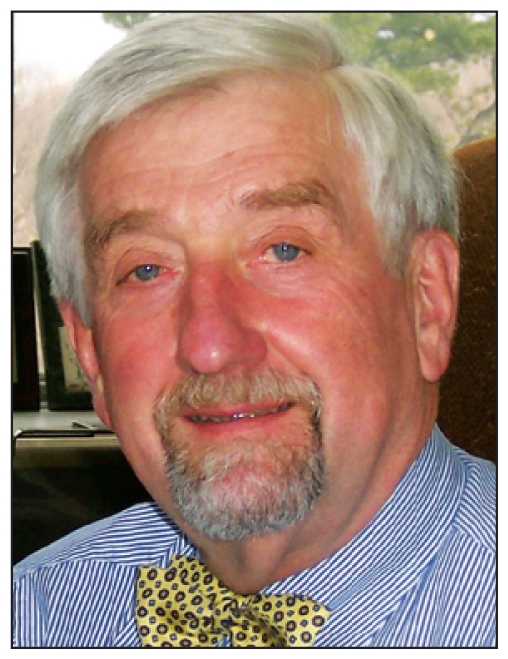
David Carpenter

**Figure:**
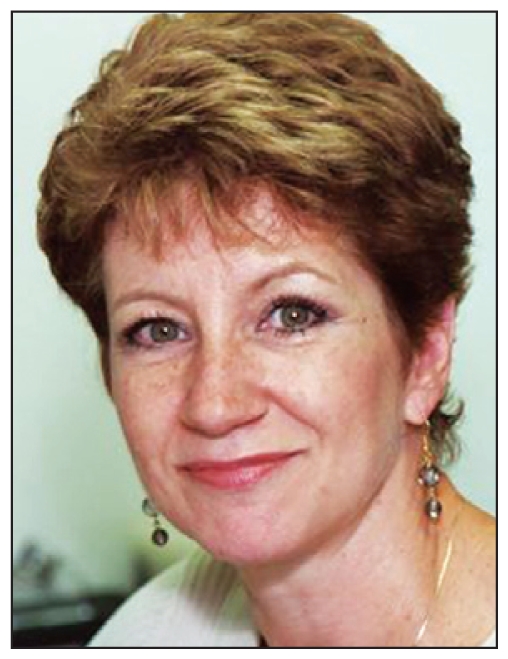
Deborah Cory-Slechta

**Figure:**
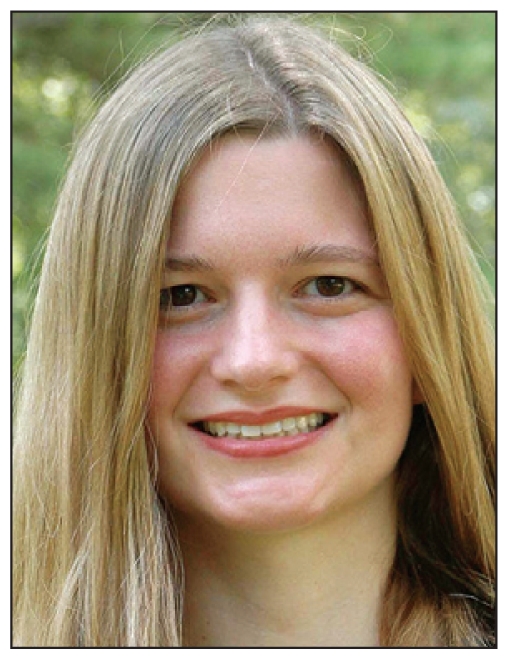
Amy Herring

**Figure:**
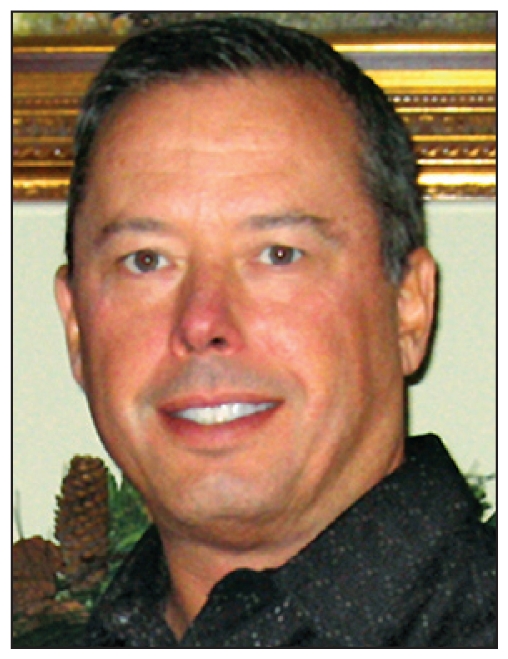
William Kelce

**Figure:**
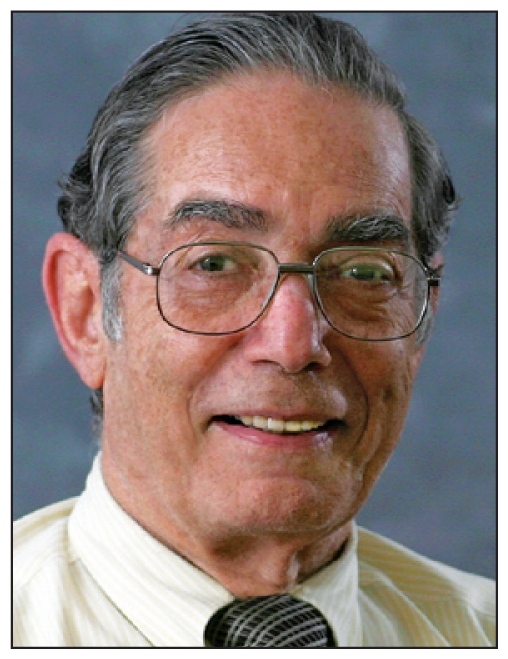
Morton Lippmann

**Figure:**
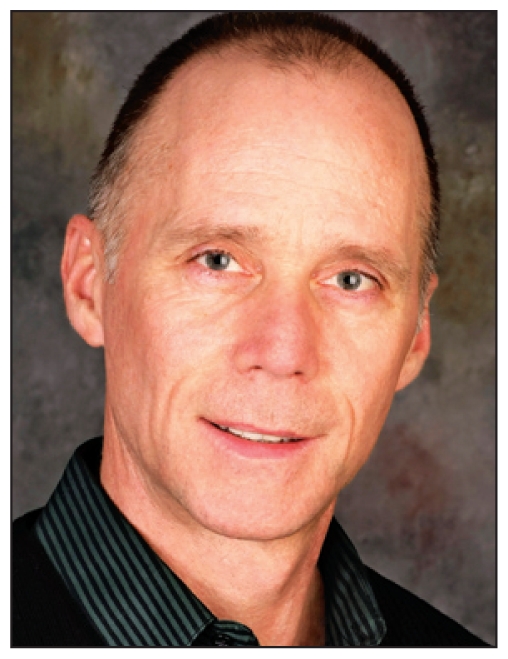
Matthew Longnecker

**Figure:**
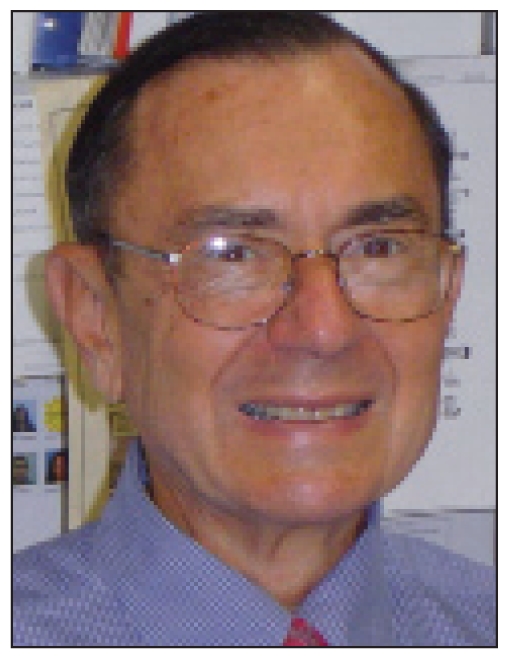
Arnold Schecter

**Figure:**
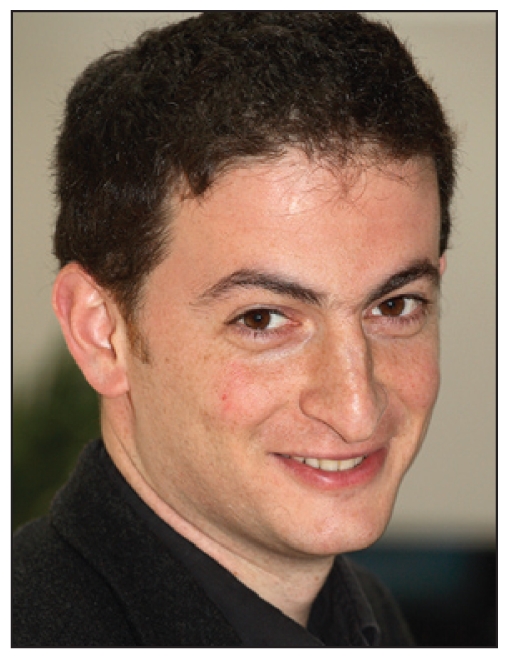
Remy Slama

**Figure:**
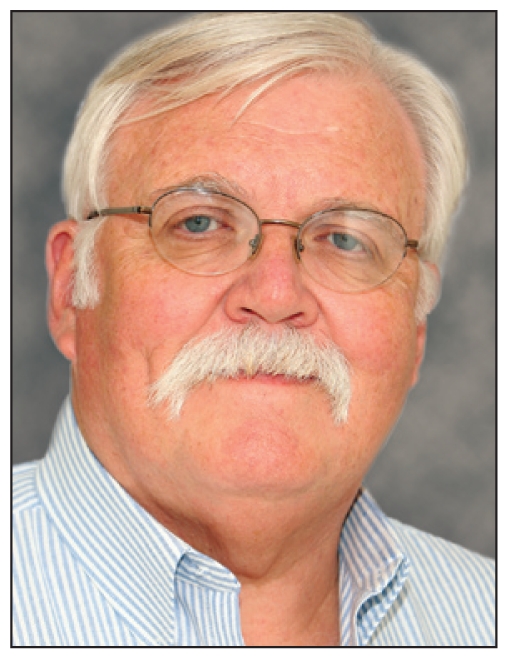
David Thomas

**Figure:**
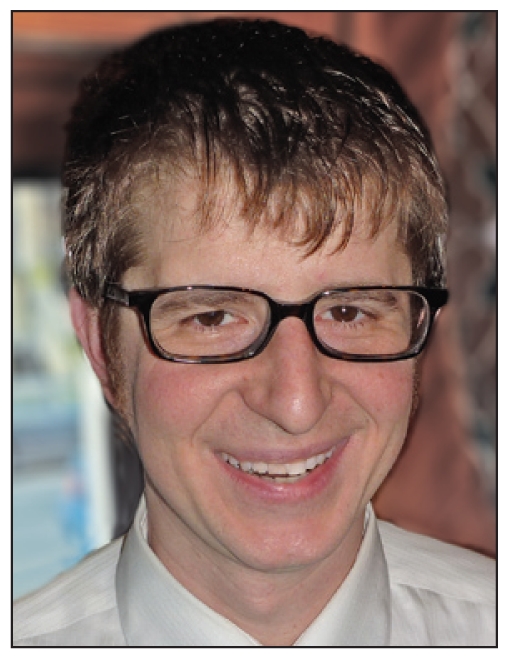
Leo Trasande

**Figure:**
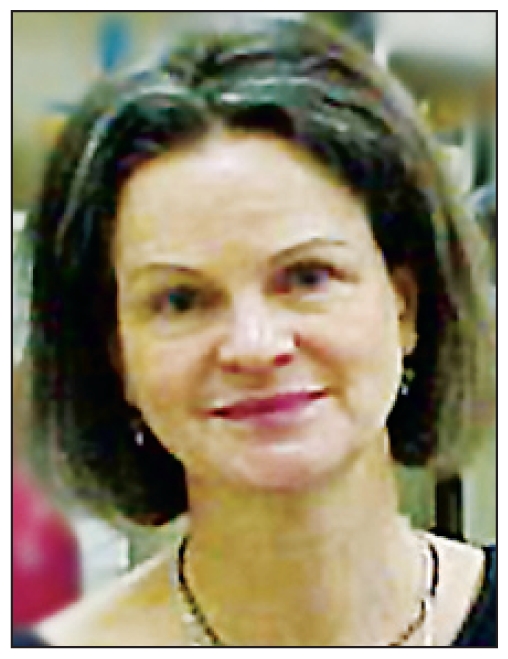
Mary Wolff

